# Orienting the Pore Morphology of Core-Shell Magnetic Mesoporous Silica with the Sol-Gel Temperature. Influence on MRI and Magnetic Hyperthermia Properties

**DOI:** 10.3390/molecules26040971

**Published:** 2021-02-12

**Authors:** Alexandre Adam, Ksenia Parkhomenko, Paula Duenas-Ramirez, Clémence Nadal, Geoffrey Cotin, Pierre-Emmanuel Zorn, Philippe Choquet, Sylvie Bégin-Colin, Damien Mertz

**Affiliations:** 1 Institut de Physique et Chimie des Matériaux de Strasbourg (IPCMS), UMR-7504 CNRS-Université de Strasbourg, 23 rue du Lœss, 67034 Strasbourg, France; alexandre.adam@ipcms.unistra.fr (A.A.); paula.duenas-ramirez@ipcms.unistra.fr (P.D.-R.); clemence.nadal@univ-tlse3.fr (C.N.); geoffrey.cotin@ipcms.unistra.fr (G.C.); sylvie.begin@ipcms.unistra.fr (S.B.-C.); 2 Institut de Chimie et Procédés pour l’Energie, l’Environnement et la Santé (ICPEES), UMR-7515 CNRS-Université de Strasbourg, 25 rue Becquerel, 67087 Strasbourg, France; parkhomenko@unistra.fr; 3 Imagerie Préclinique—UF6237, Pôle d’imagerie, Hôpitaux Universitaires de Strasbourg, 67000 Strasbourg, France; PierreEmmanuel.ZORN@chru-strasbourg.fr (P.-E.Z.); pchoquet@unistra.fr (P.C.); 4 Service de Radiologie 2, Hautepierre, Pôle d’imagerie, Hôpitaux Universitaires de Strasbourg, 67000 Strasbourg, France; 5 Icube, équipe MMB, CNRS, Université de Strasbourg, 67000 Strasbourg, France; 6 Fédération de Médecine Translationnelle de Strasbourg, Faculté de Médecine, Université de Strasbourg, 67000 Strasbourg, France

**Keywords:** mesoporous silica, magnetic nanomaterials, MRI contrast agents, magnetic hyperthermia

## Abstract

The controlled design of robust, well reproducible, and functional nanomaterials made according to simple processes is of key importance to envision future applications. In the field of porous materials, tuning nanoparticle features such as specific area, pore size and morphology by adjusting simple parameters such as pH, temperature or solvent is highly needed. In this work, we address the tunable control of the pore morphology of mesoporous silica (MS) nanoparticles (NPs) with the sol-gel reaction temperature (T_sg_). We show that the pore morphology of MS NPs alone or of MS shell covering iron oxide nanoparticles (IO NPs) can be easily tailored with T_sg_ orienting either towards stellar (ST) morphology (large radial pore of around 10 nm) below 80 °C or towards a worm-like (WL) morphology (small randomly oriented pores channel network, of 3–4 nm pore size) above 80 °C. The relaxometric and magnetothermal features of IO@STMS or IO@WLMS core shell NPs having respectively stellar or worm-like morphologies are compared and discussed to understand the role of the pore structure for MRI and magnetic hyperthermia applications.

## 1. Introduction

Among the range of inorganic nanomaterials, mesoporous silica (MS) are particularly appealing given their specific controlled pore morphology, high pore volume and large surface area [1,2,3]. In addition, their synthesis process is usually robust and scalable and silanol groups at the MS surface allow to envision the grafting of a versatile range of chemical functions [4,5,6]. Given these features, MS are promising for range of applications including catalysis [7,8], depollution [9,10] or drug delivery [11,12,13].

In general, MS are formed through surfactant-mediated assembly, which means that the first step of the process consists in the self-organization of surfactant phase, which acts as a soft template for the silica network formation [14,15,16]. This template that dictates the resulting pore size and morphology of the MS network is usually removed after silica condensation. The most reported methods to synthesize MS NPs, which are adapted historically from well-established MCM41 MS nanoparticle synthesis [17,18], make use of hexadecyltrimethylammonium bromide (CTAB), a quaternary ammonium surfactant, which in the presence of silicate precursors, typically tetraethoxysilane (TEOS), and in basic conditions, self-organizes into a hexagonal micellar phase [19,20,21]. This process results in MS NPs having an ordered hexagonal pore structure of ca. 2.5–3.0 nm pore size, corresponding to the initial diameter of the micellar rods.

To afford tunability over the pore network structures, various methods were developed to change the pore morphology and size, by playing on various synthesis parameters. Hence, increasing surfactant concentration [22] or surfactant to silica ratio allows to tune the surfactant phase nature and then the ordered pore structures [23]. The pore diameter can be modulated by varying the surfactant chain length or by using pore expandable agent such as trimethylbenzene interacting with the micellar phase [24,25,26]. The pore morphology (stellar, radial, worm-like) was also varied with the use of appropriate co-solvents during the synthesis [27,28,29]. At last, bimodal pore structures in submicron MS were also achieved by using an anionic polyelectrolyte that self-assemble with the CTAB surfactant to form macromolecular template which upon silica condensation provide two distribution of pores: one small usual pore of 2.5 nm and another of 20–50 nm pore size due to polymer and silica segregation during the sol gel reaction [30,31].

Recently, Zhang, Bonneviot et al. introduced an elegant method to finely control, in a robust and reproducible manner, the pore morphology of MS NPs having size in the range of 100 nm [32]. In this work, the authors have used small organic amines (SOA) to control the silica growth allowing hence to reach a high level of NPs monodispersity. Moreover, they showed that when MS NPs synthesis is performed in mild basic conditions (pH near 7 due to low amount of SOAs), the nature of the surfactant counterion used, determines completely the pore structure of the resulting MS NP. Indeed, in mild basic conditions, the use of tosylate anion (Tos^−^) as a counterion of hexadecyltrimethylammonium (CTA^+^) leads to weak templating promoting silica condensation around packed CTA^+^ micelles whereas the use of bromide anion (Br^−^) leads to a stronger templating resulting in an individual coverage of the micellar rods by silica around. Hence, in these conditions, CTA^+^ templating with Tos^−^ orientates the pore morphology towards stellar large pore structure (10–15 nm pore size) while Br^−^ orientates towards small pores structures resulting in so-called raspberry structures. Furthermore, when the concentration of the SOA is increased, the authors reported that the counterion is no longer the factor determining the pore structure and a new phase called worm-like (WL) is formed. This latter supposes high level of surfactant interpenetration during silica condensation. Despite the wide range of parameters investigated, the authors achieved the synthesis only at a reaction temperature set at 80 °C and the influence of this fundamental parameter was to the best of our knowledge not reported for such MS structure. However, modulating this parameter experimentally would be very easy to achieve, and changing the pore morphology by this way would afford a new controlled and simplified process leading to new structural features on MS NPs but also on MS shell deposited at the surface of inorganic cores such as carbon nanotubes CNTs, iron oxide (IO), or gold NPs, which are well-known to be activable by external fields. 

In our previous works, we have recently developed various functionalization strategies using synthesized stellate MS NPs to develop innovative nanomaterials for luminescence bioimaging in vivo [33] or efficient chelating metal capture in a biological media [34]. MS stellate shells were also deposited around iron oxide NPs for combined imaging and hyperthermia applications [35] or to ensure formulation of a new generation of magnetic glasses [36]. In all these examples, the stellate morphologies were performed at the same temperature synthesis set at of 75–80 °C, which determine similar stellar morphology and pore size.

**Scheme 1:**
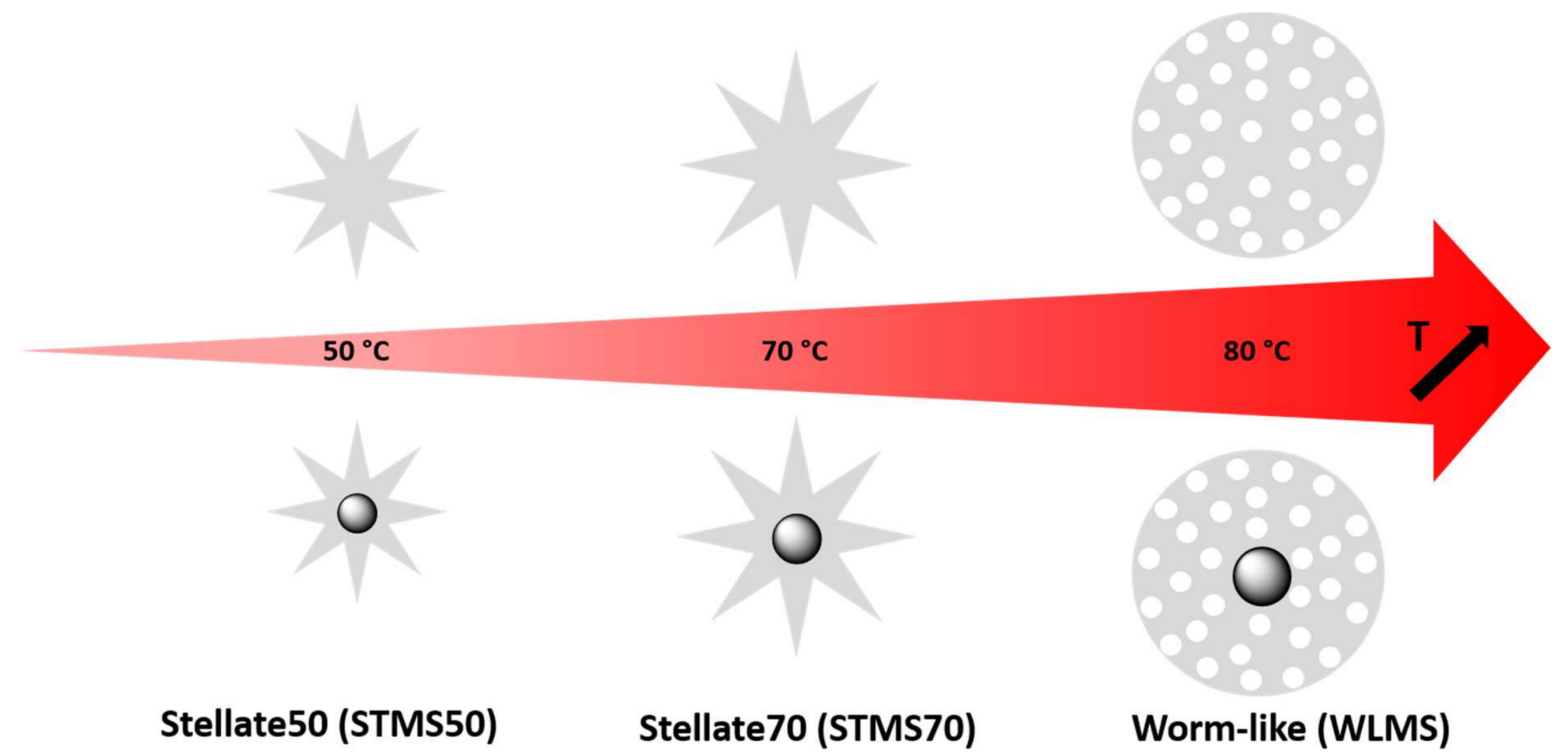
Scheme illustrating the thermally controlled process to orient the pore morphology of mesoporous silica (MS) and IO@MS NPs from stellate (STMS) to worm like (WLMS).

Herein, the influence of the temperature as a simple synthesis parameter was investigated with the aim to modulate the pore structure morphology of MS NPs and MS shell coated around IO core. We report here that above a certain threshold of the sol gel reaction temperature (T_sg_) measured at around 80 °C the pore structure is found to change from a stellar (ST) to a worm-like (WL) morphology (Scheme 1).

Hence, in a first part of this work, the reaction temperature during the MS NPs synthesis in precise conditions (CTATos used as a surfactant, in mild basics conditions pH 7, with TEOS as the silica source) was hence varied in the range of 50 to 85 °C. The resulting MS NPs size distribution, morphology and pore structure were carefully characterized by transmission electron microscopy (TEM) and N_2_ adsorption-desorption isotherm to understand the structural changes and decipher micro and mesoporosity. In a second part, we carried out this reaction at two different temperatures in the presence IO NPs, of 18 nm diameter, made by thermal decomposition, to investigate the possibilities to obtained magnetic core shell having a MS shell with a tuned pore structure, namely IO@STMS or IO@WLMS morphology.

With the aim to highlight the potential of such magnetic core shell as T_2_-weighted MRI contrast agents, the relaxometric properties of the IO@STMS or IO@WLMS are investigated by measuring the longitudinal and transversal relaxivities. In order to emphasize their potential as magnetic nanoheaters for magnetic field induced hyperthermia, temperature profiles under an alternating magnetic field (AMF) are traced and measurements of the specific absorption rates (SAR) are achieved and compared to discuss on the influence of the pore morphology on magnetically induced heat dissipation useful for magnetic hyperthermia treatment.

## 2. Results and Discussion

**Figure 1:**
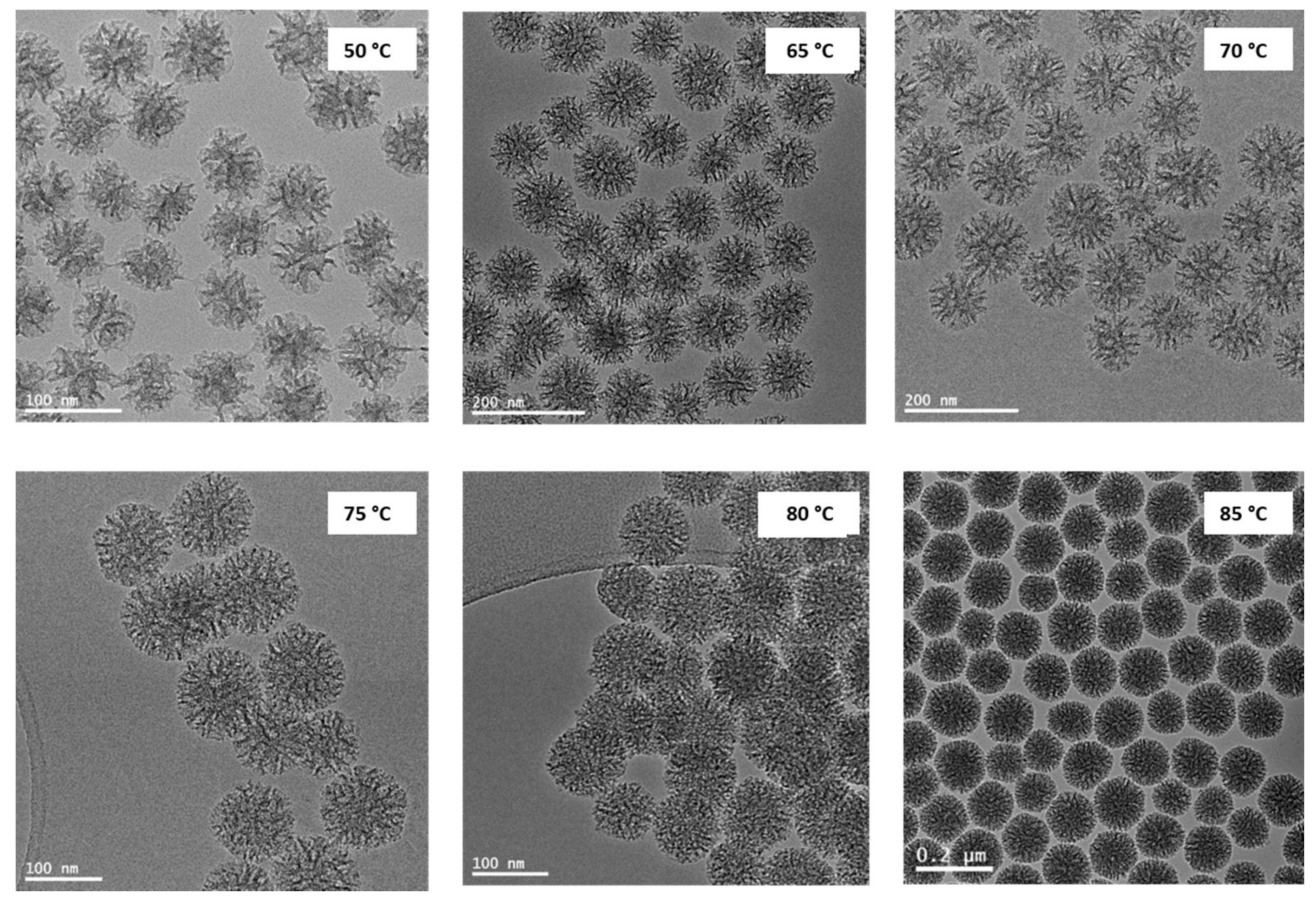
TEM images of MS NPs for different sol-gel reaction temperature: 50 °C, 65 °C, 70 °C, 75 °C, 80 °C and 85 °C. Below 75 °C, the MS present ST pore morphology whereas above 80 °C, WL type appears.

In a first study, the reaction temperature of the CTATos-mediated sol-gel MS NPs synthesis was modulated from 50 °C to 85 °C. For STMS NPs synthesis, the CTATos surfactant solution was heated up to the desired temperature (<80 °C) and TEOS was added without delay to the reaction flask. Concerning WLMS NPs, the temperature was set above 80 °C and the solution was stirred more than one hour before TEOS addition. 80 °C was found as a frontier temperature: Below it, STMS were formed and above it, WLMS NPs were obtained. That is why a dwell time of one hour before the introduction of the TEOS silica precursor at T ≥ 80 °C was added in order to let the time for the WL phase to stabilize and thus get very reliable syntheses. Without this delay, it happened that some intermediate phases between ST and WL were produced. It is important to note that adding a dwell time below 80 °C has no influence and it usually leads to stellate morphology. Finally, surfactant was removed by calcination and TEM imaging was performed on the MS NPs obtained at six different temperatures: 50, 65, 70, 75, 80 and 85 °C (see Figure 1).

TEM images show that a change of the pore morphology appears from 75 °C to 85 °C. Below 75 °C, NPs have a stellate morphology whereas above 80 °C smaller pores are clearly observed. The average diameter of the MS NPs was measured as a function of the temperature (see Figure S1). For STMS, the size increases from 70 ± 7 nm at 50 °C to 105 ± 10 nm in the range of 65 to 75 °C. WLMS are even bigger, around 120 ± 12 nm when they are synthesized at 85 °C. Overall, the NPs diameter increases with the reaction temperature, indicating that the condensation of the silica precursor, TEOS, is favored at higher temperatures. Observation of the TEM images indicates that the as synthesized MS NPs display a narrow size distribution.

**Figure 2:**
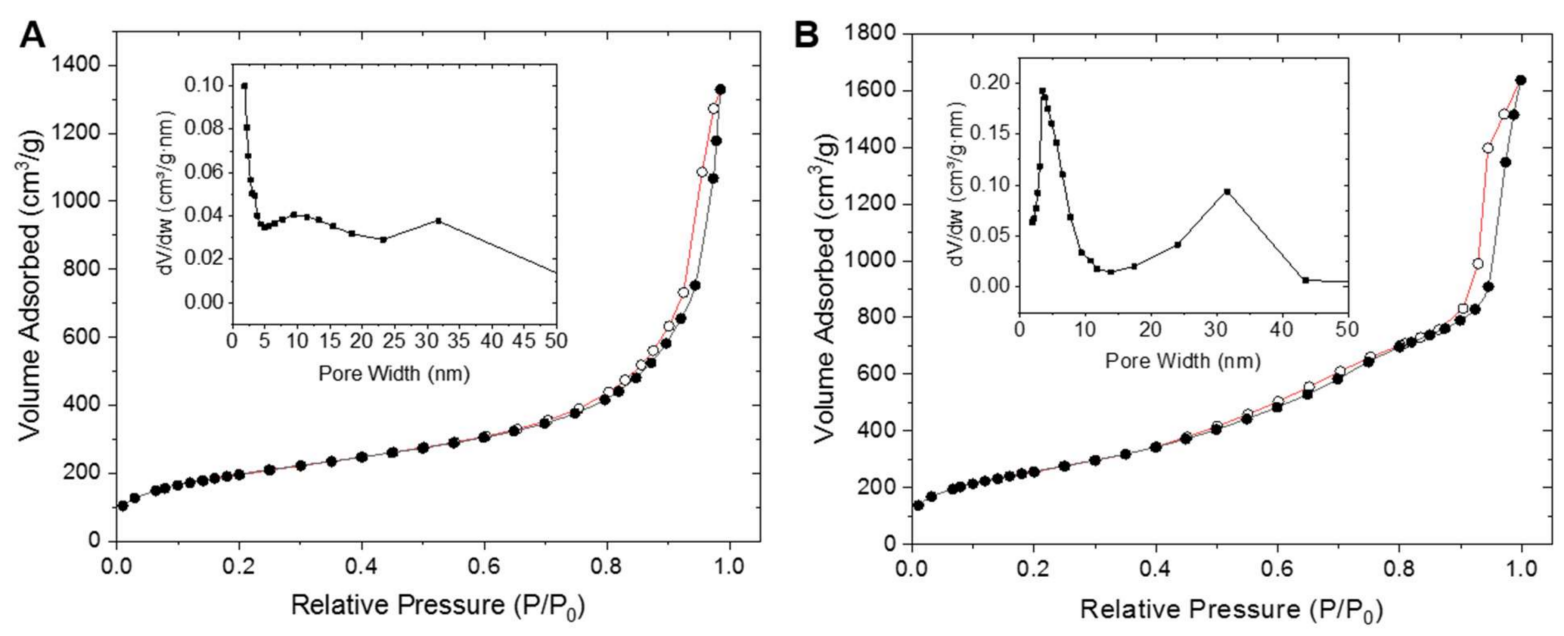
Adsorption-desorption isotherms at high relative pressures for the samples STMS50 (**A**) and WLMS80 (**B**) including Barrett–Joyner–Halenda (BJH) desorption pore volume plots for STMS50 (inset **A**) and WLMS80 (inset **B**).

N_2_ adsorption-desorption isotherms were performed with the aim to analyze the pore structure of the MS NPs, especially their mesoporosity and microporosity in correlation with the TEM images. Stellate NPs obtained at 50 °C (STMS50) were investigated and compared with WLMS NPs synthesized at 80 °C (WLMS80) (Figure 2).

The BET method of the studied samples indicates specific surface areas of 713 m^2^ g^−1^ for STMS50 and 950 m^2^ g^−1^ for WLMS80. The form of the isotherms at high relative pressure (Figure 2A,B) shows the micro-mesoporous character of the samples (type IV). The sharp uptake on these isotherms at the relative pressure of around 0.8–1.0 is due to the small particles nanoscale and corresponds to the interparticle adsorption or void volume between particles. It is difficult to predict the shape of the mesopores because the hysteresis loops are not representative, but it is possible to confirm that the materials have micro-mesoporous morphology—long, flat adsorption with small hysteresis loop [37]. Indeed, the mesopores presence in STMS50 was evidenced by the presence of two small peaks on the Barrett–Joyner–Halenda (BJH) desorption pore size distribution plot (inset Figure 2A) corresponding to 10 nm (mesopores of the STMS50) and 30 nm (interparticle voids). Then, by increasing the sol-gel reaction temperature from STMS50 to WLMS80 a change in the textural properties is clearly observed by the BJH desorption pore size distribution plots. Figure 2B shows that the mesopore size distribution stays bimodal with 4 nm (mesopores of the WLMS80) and 30 nm (interparticle voids). Hence, the majority of the 10 nm mesopores in STMS50 disappeared and is replaced by 4 nm mesopores in WLMS80. Pore volume of mesopores (V_meso_, Table 1) was calculated by integration of the adsorbed volume of nitrogen from BJH pore volume distribution plot including only the pores from 2 to 15 nm.

**Figure 3:**
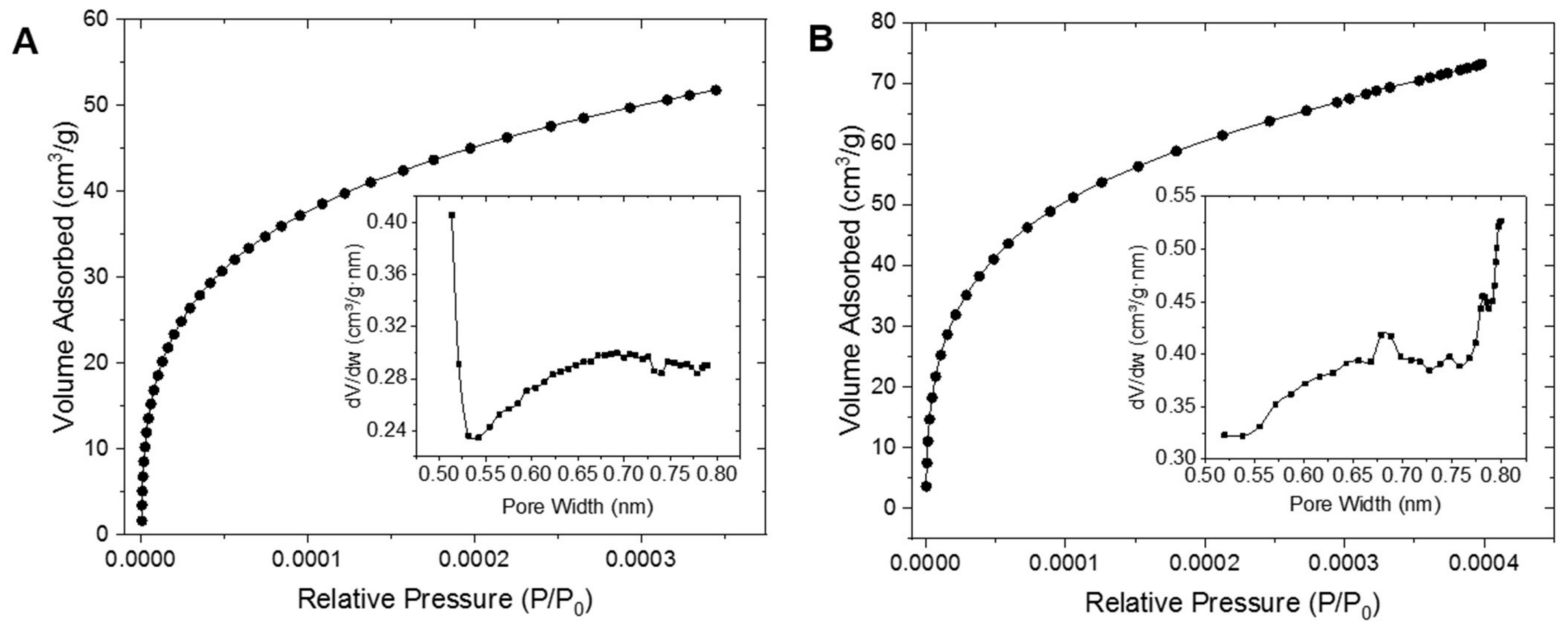
Adsorption isotherms at small relative pressures for STMS50 (**A**) and WLMS80 (**B**) including Horvarth–Kawazoe differential pore volume plots for STMS50 (inset **A**) and WLMS80 (inset **B**).

The isotherms at low relative pressure (Figure 3A,B) correspond to microporous character of the materials (Type I). V_micro_ includes only micropores and was calculated by integration of the adsorbed volume of nitrogen from Horvarth–Kawazoe differential pore volume plot and it corresponds absolutely to the pore volume calculated at the single point of the relative pressure equal to 0.0004 (Table 1). On the pore size distribution of the STMS50 material, it is clear that STMS50 has a big quantity of very small micropores with the maximum pore size around 0.5 nm (inset Figure 3A). Regarding WLMS80, the most present micropores size switched from 0.5 to 0.8 nm and the quantity of mesopores largely increased (Figure 2B and Figure 3B).

The calculated ratio between pore volumes corresponding to mesopores and to micropores (see Table 1) shows an increase of mesoporosity due to the treatment at 80 °C. Indeed, the V_meso_/V_micro_ ratio increased from 6.6 to 8.8. The increased temperature probably collapsed small micropores of ≤0.5 nm size in favor of the micropores with bigger size that tend to mesoporosity. It is visible in the comparison of Figure 3A,B (pore size distribution plots) that the volume of micropores did not decrease but the distribution of pores size tends to switch to bigger values. Increasing the synthesis temperature probably also densified the walls of the bigger pores thus creating more mesoporosity that is in correspondence with the increased specific surface area and slight increase of the micro- and mesopores volumes. For both cases, the volume of mesopores represents the largest part of the total pore volume (Table 1). This confirms their crucial importance as the mesopores are mainly on the surface of the NPs, interacting with their environment.

For a reaction temperature under 75 °C stellate phase MS NPs are produced. Their formation follows certainly the mechanism proposed earlier by Zhang et al. [32]. The tosylate counterion (Tos^−^) plays a major role here in the structural organization of the CTA^+^ micelles. Tos^−^ competes against the adsorption of the silicate oligomers forming through the hydrolysis/condensation of TEOS in the presence of an organic base (AHMPD). The CTA^+^ micelles, which are partially silicated, are then pushed together to self-assemble into bigger bundles. These latter lead to the described big mesoporosity around 10 nm after surfactant removal. From our experiments, this process is valid in a range between 50 °C to 75 °C. Above 80 °C, this kind of self-assembly does not occur anymore. The micellar rods seem to interpenetrate each other certainly due to thermal agitation. By raising the reaction temperature, the hydrolysis/condensation rate of TEOS is increased. Consequently, the density of silanolates in the reaction solution is increased, which results in a displacement of the equilibrium towards a better coverage of the surfactant micelles by silicate. Self-assembly into big bundles is thus prevented. The increase of the temperature could also destabilize the Tos^−^/CTA^+^ micelle electrostatic interaction allowing the silicate oligomers to condense preferentially around the micelles. Both phenomena would promote stronger templating conditions that leads to a worm-like organization of the pore channels. Thus, big pores of 10 nm diameter in STMS disappear and are replaced by smaller 4 nm pores in WLMS.

**Scheme 2:**
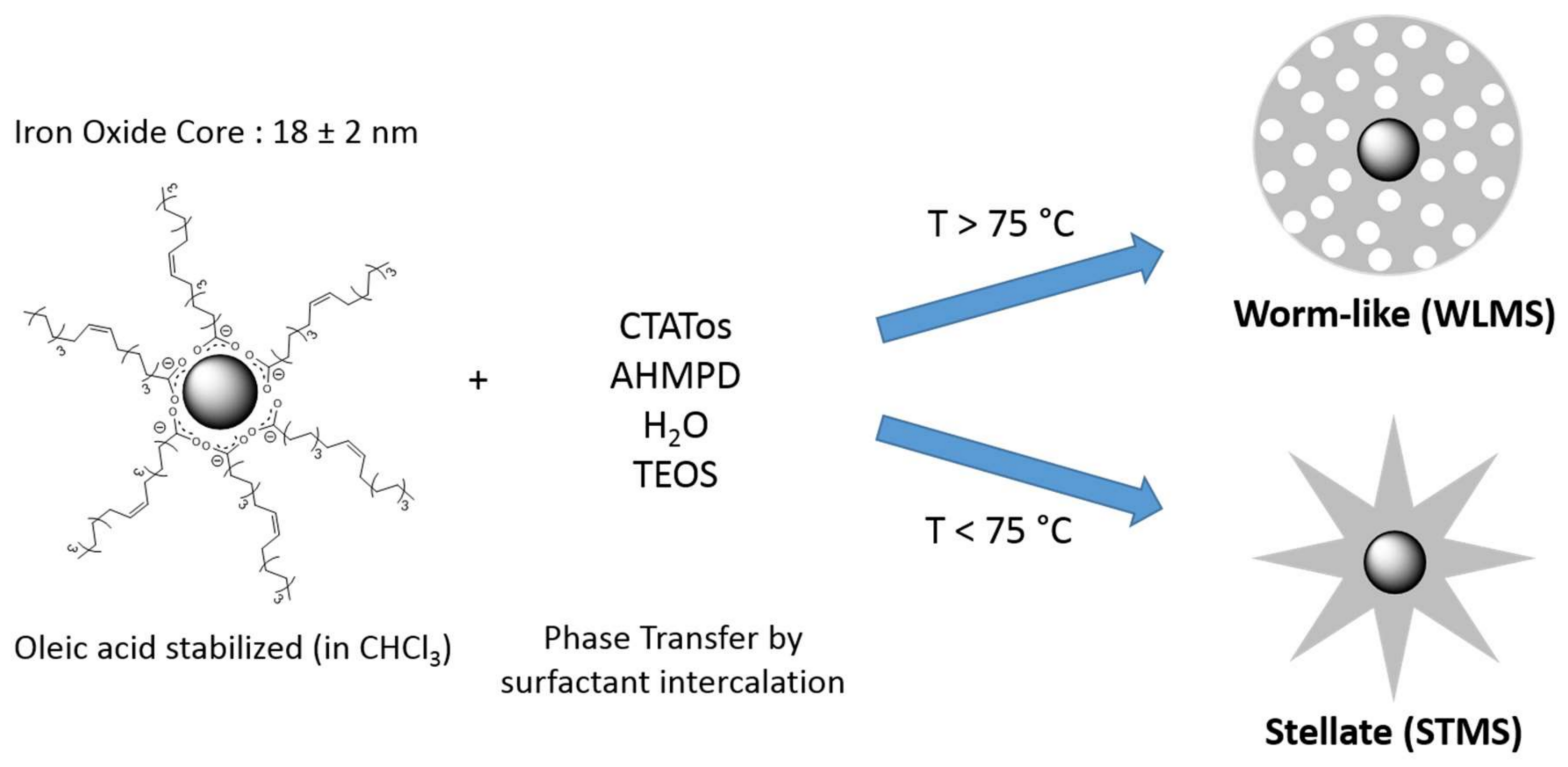
Coating of IO NPs with stellate (STMS) or worm-like mesoporous silica (WLMS) morphologies.

Furthermore, the coating of inorganic NPs with silica is very attractive as it improves the stability of the cores, it brings a chemically versatile surface and leads to a very good colloidal stability in aqueous environments. Hence, in a second study, the previously described approach was transposed to the coating of magnetic iron oxide cores (IO NPs) as pictured in Scheme 2. The influence of the silica morphology was then studied.

**Figure 4:**
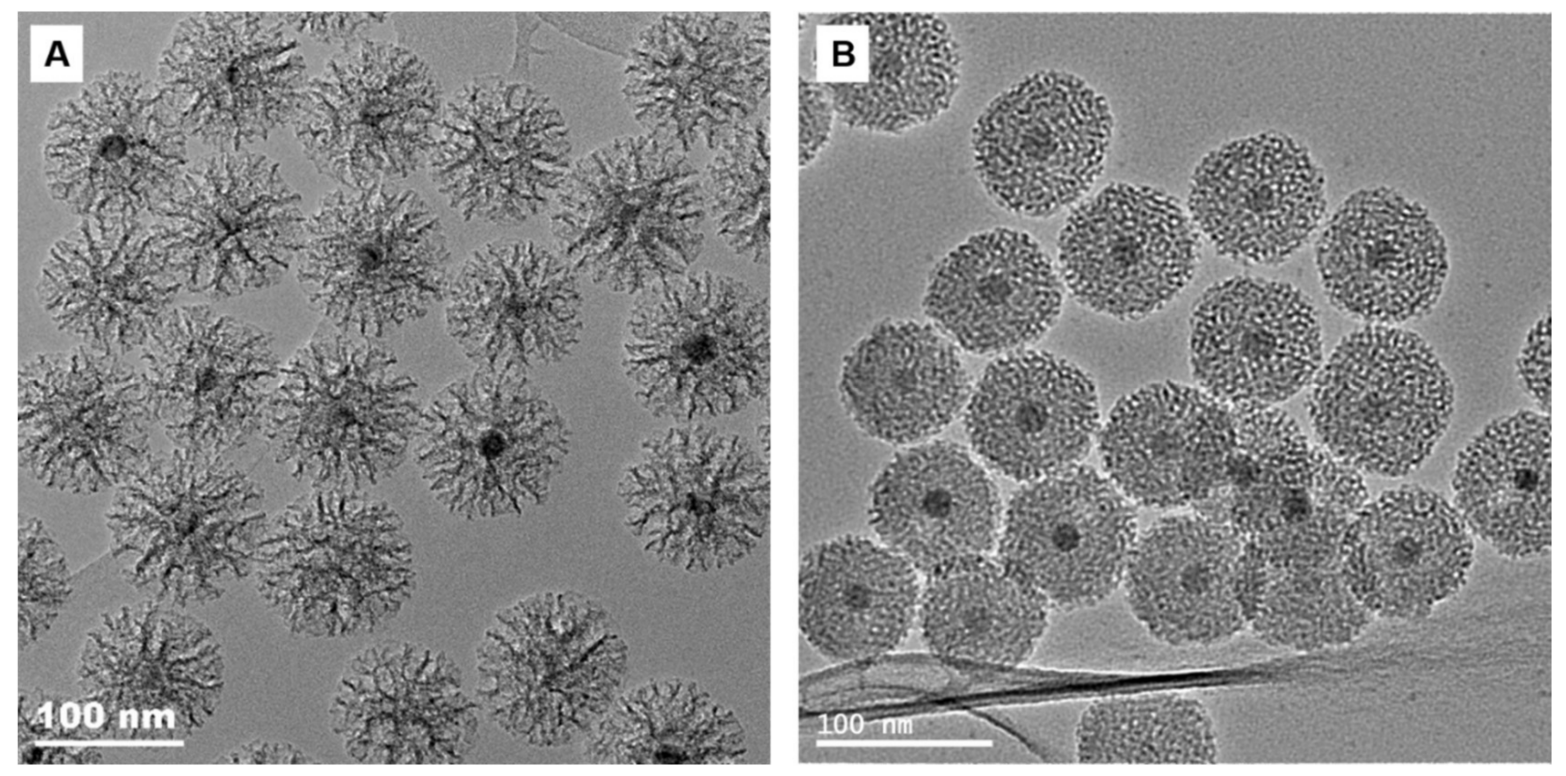
IO@STMS synthesized at 70 °C (**A**) and IO@WLMS synthesized at 80 °C (**B**).

Spherical oleic acid-stabilized iron oxide NPs synthesized by thermal decomposition of ca. 18 nm diameter are known to have superparamagnetic behavior at room temperature. Such 18 nm IO NPs were shown to be suitable for magnetic hyperthermia and MRI applications [35,38,39,40]. MS shells of various morphology were thus formed around magnetic core redispersed in chloroform by phase transfer in CTATos surfactant aqueous solution. Similar procedures as described above without magnetic core were carried out at 70 °C and 80 °C. After washing and surfactant extraction steps, TEM images were performed (Figure 4). TEM images show that the previous protocol is applicable and transposable to the coating of oleic acid-capped inorganic NPs. Stellate morphology (IO@STMS) was obtained at sol gel temperature of 70 °C and worm-like morphology at 80 °C. The synthesized NPs presented a very good homogeneity with very few NPs without IO core and a narrow size polydispersity: 100 ± 10 nm for IO@STMS and 75 ± 6 nm for IO@WLMS. Even if it is assumed that the mechanisms of the silica condensation and pore morphology formation in presence of IO cores and without core are very similar, it is noted that IO@WLMS display a smaller diameter than without IO cores as seen above. This is more likely due to the slight variation of the synthesis ratio IO cores/TEOS/surfactant than by the effect of the temperature itself. Iron oxide playing the role of seeds here, this difference of parameters governing silica formation may induce different resulting diameters in the presence or in absence of IO cores.

We expected here that the pore morphology had an important impact on the environment around the magnetic core. The organization and the structure of the silica pores (meso and micro) should thus influence the relaxometric and magnetothermal properties of the nanocomposites IO@MS NPs [41,42] as observed in previous works. Hence to address these questions, the relaxometric and magnetothermal properties of these materials were evaluated for the two morphologies: IO@STMS and IO@WLMS.

**Figure 5:**
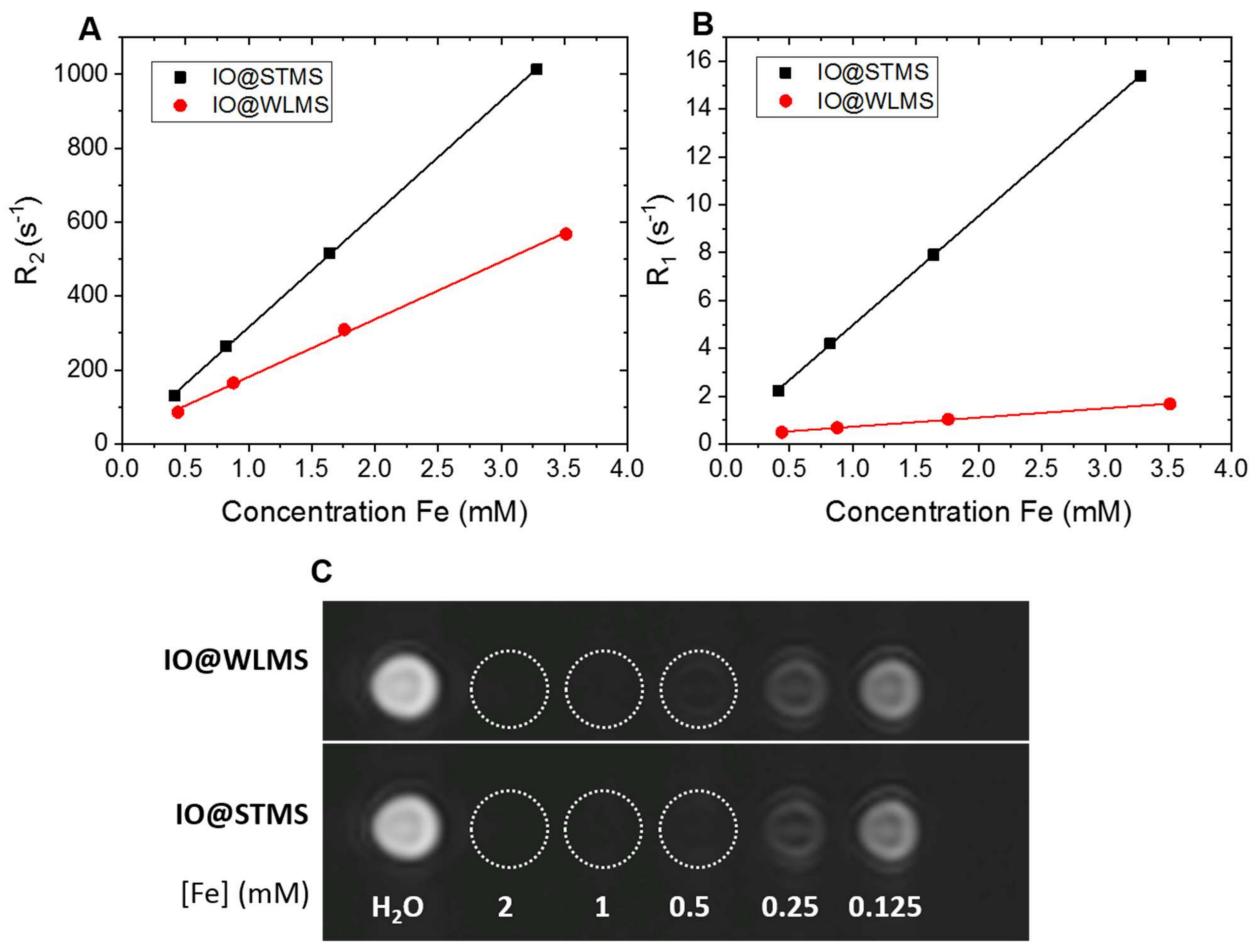
Transversal R_2_ = 1/T_2_ (s^−1^) relaxation rates (**A**) and longitudinal R_1_ = 1/T_1_ (s^−1^) relaxation rate (**B**) as a function of [Fe] (mM) for IO@STMS and IO@WLMS in water at 37 °C and 1.41 T; T_2_-weighted (**C**) MRI of IO@WLMS and IO@STMS, white, dotted-line circles indicate sample positions.

IO NPs are well known as hypocontrast T_2_-weighted MRI contrast agents. Transversal and longitudinal relaxation times of water protons (T_2_ and T_1_ respectively) were recorded as a function of the iron concentration, i.e., as a function of the core-shell NPs concentration, for the two silica pore morphologies (STMS and WLMS). Figure 5 shows the variation of the relaxation rates R_2_ = 1/T_2_ and R_1_ = 1/T_1_ as a function of [Fe]. The graphs indicate linear profiles and the slope corresponds to the transversal relaxivity (r_2_) and longitudinal relaxivity (r_1_). Results indicate that IO@STMS has a transversal relaxivity twice higher than IO@WLMS (307 mM^−1^·s^−1^ vs. 156 mM^−1^·s^−1^) (Table 2). This suggests that the diffusion of water molecules in and around the silica shell is better with a stellate morphology as compared to the worm-like one. Regarding longitudinal relaxivity r_1_, the measured value for IO@STMS is more than ten times higher than r_1_ of IO@WLMS. For the same IO core, such a higher value implies that water molecules have an enhanced accessibility to the central core when the pore morphology is stellate. Moreover, the MRI contrast of the solutions were tested on a 3 T clinical instrument. On Figure 5C, phantom images show a strong T_2_-weighted hypocontrast effect for both pore morphologies. This hypocontrast of the nanocomposites is very strong and complete darkening is observed already at low iron concentrations—almost no more signal at 0.5 mM. IO@STMS show a slightly highest hyposignal than IO@WLMS, which is in agreement with the previous relaxivity measurements.

Overall, these results show that the silica pore morphology have an impact on the MRI imaging properties. Stellate-like pore organization in IO@STMS allows a better water accessibility and diffusion in the nanocomposites compared to IO@WLMS. It can be rationalized that the very large open pores of the STMS shell is the key factor explaining this behavior. These effects on the relaxivities are in agreement with behaviors previously encountered with iron oxide@silica shell having micro porosities or small mesopores.

Furthermore, when superparamagnetic IO NPs are placed in an alternating magnetic field (AMF), heat is generated and released in the surrounding medium. This heat (magnetothermal transfer) comes from the Brownian and Néel’s spin relaxations [38,43,44]. A coating of a porous shell around IO core is a way to bring new features to the magnetic core: Colloidal stability, high level of chemical functionalization or drug loading [35,45,46]. As seen above in the relaxometric study, this MS shell have an influence on the properties of the core, and the magnetothermal properties as a function of the pore morphology are also investigated as follows.

**Figure 6:**
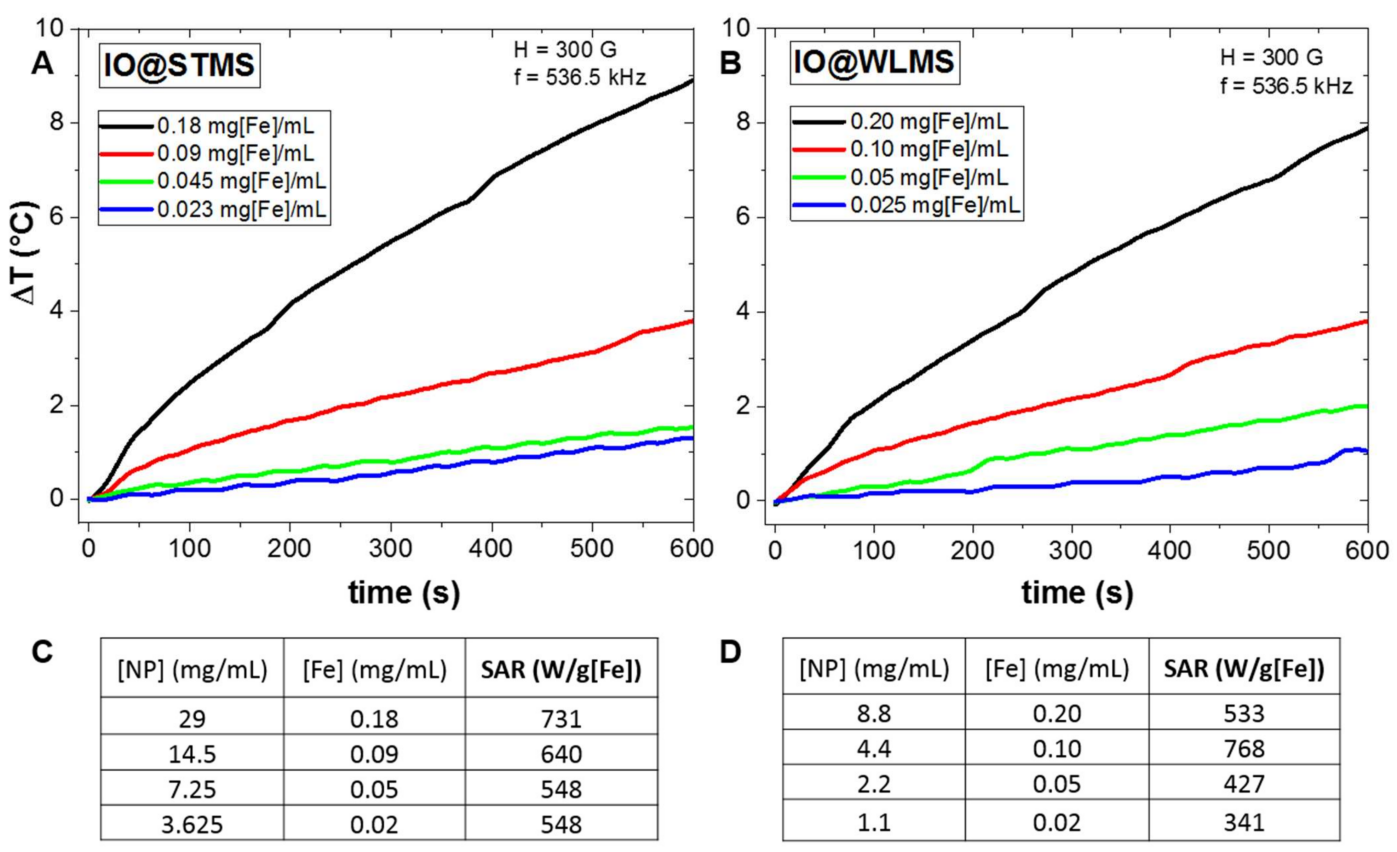
Temperature profiles as a function of time under alternating magnetic field (AMF) for different concentrations of IO@STMS (**A**) and IO@WLMS (**B**) including the corresponding SAR values for IO@STMS (**C**) and IO@WLMS (**D**).

Calorimetric measurements allow to evaluate the heat power dissipated by the NPs (per mass unit of material), which is also called the specific absorption rate (SAR). The temperature profiles as a function of time and SAR values acquired at the field parameters of H = 300 G (23.8 kA.m^−1^) and f = 536.5 kHz for different concentrations of nanocomposites were studied and are shown in Figure 6. SAR were calculated following the procedure described in Materials & Methods section.

First, for both types of magnetic core shell, temperature profiles curves (Figure 6A,B) show a progressive temperature elevation of the samples dispersed in water. The measuring system is not perfectly adiabatic and some heat is lost to the environment—that is why the profiles do not describe perfect straight lines but have a damped profile. This is taken into account for the fitting of the curves and the SAR calculations [43,47,48]. For both samples, the concentration of iron oxide has a clear impact on the heating under AMF. The SAR values increase with the concentration of IO@MS (Figure 6C,D). A temperature elevation of more than 8 K can be achieved in 10 min at low iron concentration (0.18–0.20 mg[Fe]/mL). As expected by the calculation formula, SAR values should be independent from the magnetic material concentration. Here slight fluctuations of SAR are observed. Even if the MS coating weakens the dipolar interactions, they could be attributed to partial aggregation of NPs or at least by different dispersion states between the samples when AMF is applied.

Nevertheless, with SAR values in the range of [548, 731] W/g[Fe], and [341, 768] W/g[Fe] for IO@STMS and IO@WLMS core shell, respectively, it is clear that these nanocomposites are very good and performant objects to deliver heat locally with SAR suitable and adapted to perform hyperthermia treatments or heat-triggered drug delivery [39,41,42,49]. Other works reported SAR performances even for lower magnetic fields and frequencies suitable for biological applications [33,38,39,50,51,52]. From the tables in Figure 6, it is important to note that the heat dissipation ability of IO@WLMS is slightly lower than the IO@STMS though. This can be attributed to the pore morphology. The worm-like pores are less open towards the exterior of the particle and the silica is denser. Heat diffusion is thus slowed down. In addition, the synthesized IO@WLMS NPs have a smaller diameter than IO@STMS (75 nm vs. 100 nm), which means a thinner silica shell. We can assume that if the silica shell thickness of both morphologies would be equal, the damping effect of the IO@WLMS in comparison with IO@STMS would be even more pronounced.

## 3. Materials and Methods

### 3.1. Chemicals

Tetraethyl orthosilicate (TEOS, ≥ 99.0%), 2-amino-2-(hydroxymethyl)-1,3-propanediol (AHMPD, ≥ 99.9%), cetyltrimethylammonium tosylate (CTATos, ≥ 98.0%), ammonium nitrate (NH_4_NO_3_), ferric chlorid (99%) and squalane (96%) were obtained from Sigma–Aldrich (Lyon, France). Nitric acid 65% (HNO_3_) was purchased from Carlo-Erba (Barcelona, Spain). Sodium stearate (98.8%) was obtained from TCI (Tokyo, Japan). Oleic acid (99%) was purchased from Alfa Aesar (Lancashire, UK) while dibenzylether (DBE, 99%) was purchased from Acros Organic (Waltham, Massachusetts, USA).

### 3.2. Synthesis of ST and WL Mesoporous Silica Nanoparticles (MS NPs)

The protocol was adapted and modified from Zhang et al. [32]. In a typical procedure, CTATos (3.8 g, 8.3 mmol), AHMPD (436 mg, 4.15 mmol) and distilled water (200 mL) were added into a 500 mL Erlenmeyer flask. For full dissolution, the mixture was heated up to a defined temperature depending on the desired morphology. For stellate morphology (STMS) the temperature is set between 50 °C and 70 °C. Once the set temperature is reached, TEOS (3.25 mL) is immediately added. In order to get worm-like morphology (WL), the mixture is heated above 80 °C and stirred at least 1.5 h. The silica precursor TEOS (3.24 mL, 2.15 mmol) is then added. After TEOS addition, the mixture was stirred for 2 h. The NPs were then collected by centrifugation (12,000× *g*, 12 min) and calcinated at 550 °C for 6 h to remove the surfactant and any organic material. Finally, the MS NPs were crushed and dispersed in ethanol for further use and conservation.

### 3.3. Synthesis of IO Core—MS Shell Nanoparticles (IO@STMS and IO@WLMS)

Oleic acid-stabilized iron oxide nanospheres with a mean diameter around 18 nm have been synthesized by thermal decomposition following a recently reported procedure [53]. Briefly, iron stearate (III) was prepared by precipitation of sodium stearate and ferric chloride salts in an aqueous solution as described [54]. The synthesized iron(III) stearate (1.85 g, 2 mmol) was mixed with oleic acid (1.89 g, 6.7 mmol) in squalane (15.8 g, 19.5 mL) and DBE (0.53 g, 0.5 mL) in a two-neck round-bottom flask. The mixture was heated under stirring to 120 °C and kept at this temperature for 60 min. The condenser was then connected to the flask, and the solution was heated to 330 °C and kept under reflux for 60 min under air. After cooling to room temperature, the viscous suspension was solubilized in chloroform (10 mL). The NPs were precipitated by addition of an excess of acetone and washed three times with chloroform and acetone (ratio 1:4) and centrifuged at 14,000 rpm for 5 min. The NPs were redispersed in chloroform and stored until further use.

To incorporate the iron oxide core, the previous protocol was slightly adapted in order to achieve the phase transfer of IO NPs from chloroform to the aqueous phase. CTATos (192 mg), AHMPD (22 mg) and distilled water (20 mL) were added into a 50 mL Erlenmeyer flask. In order to get STMS morphology, the mixture was brought to 65 °C and then 5 mg of IO NPs dispersed in chloroform were added under vigorous stirring. The mixture was let to stir for at least 20 min. The color of the dispersion changed from hazy grey after addition to limpid dark black after full chloroform evaporation. Then the silica precursor TEOS (1.6 mL) was added and the sol-gel reaction starts. Ten minutes after TEOS addition the temperature was set to 70 °C and the mixture was stirred for 2 h.

For the WLMS morphology, the same mixture of CTATos, AHMPD and distilled water was heated up to 80 °C. Then 8 mg of IO NPs in chloroform were added and the reaction medium was stirred for 20 min. The sol-gel reaction starts after TEOS (400 μL) addition. The mixture is stirred for 2 h. NPs were then collected by centrifugation (12,000× *g*, 12 min) and dispersed in water:EtOH (1:1). CTATos extraction from the silica pores was done by mixing the NPs with NH_4_NO_3_ (20 mL, 20 mg.mL^−1^ in EtOH) followed by stirring and heating at 60 °C during at least 1 h. The CTATos extraction was done several times and followed by zeta potential analysis. After the synthesis, the NPs have a zeta potential above +30 mV at pH = 7. With surfactant extractions, it decreases until it reaches a plateau around −20 mV. Finally, the NPs were dispersed in EtOH, were denoted IO@STMS or IO@WLMS NPs and were stored at room temperature until further use.

### 3.4. Characterization Techniques

#### 3.4.1. Transmission Electron Microscopy (TEM)

The MS NPs and IO@MS NPs were deposited on carbon-coated copper grids. TEM images were acquired with a JEOL 2100 TEM instrument (JEOL Ltd, Tokyo, Japan) operating at 200 kV. The software Image J (NIH, Bethesda, MD, USA, public domain) was used to determine the size distribution of the NPs.

#### 3.4.2. N_2_ Adsorption Desorption Isotherms

The textural properties of the prepared samples were studied by N_2_ adsorption-desorption measurements at −196 °C. The nanoparticles were degassed under vacuum at ambient temperature (around 20 °C) for 3 h to desorb the moisture before analysis. Specific surface area was calculated by Brunauer–Emmet–Teller (BET) method. Pore volume and pore distribution were determined using desorption branch by the Barrett–Joyner–Halenda (BJH) method. Horvath-Kawazoe model was used for determining pore-size distribution in a micropore analysis from a single adsorption isotherm (dosing of nitrogen 2 cm^3^, stability time 3 h).

#### 3.4.3. Iron Dosage by NMR ^1^H-Relaxometry

The amount of iron in the NPs was quantified by *T*_1_ relaxation time measurements. Previously, a calibration curve was established by measuring the longitudinal relaxivity *r*_1_ of a standard solution of iron III nitrate at 2 wt% HNO_3_. This allows to plot the variation of the relaxation rates (1/*T*_1_) as a function of [Fe^3+^] from 0 to 3.6 mmol.L^−1^. The IO@MS nanocomposite suspension was digested with concentrated nitric acid (65 wt%) until full dissolution of iron oxide. Moderated heating at 60 °C could be used to accelerate the digestion. The sample is then diluted to reach 2 wt% HNO_3_ and the *T*_1_ relaxation time was measured and compared with the calibration curve to determine the iron content.

#### 3.4.4. Relaxivity Measurements

The measurements of longitudinal *T*_1_ and transversal *T*_2_ relaxation times of IO@STMS and IO@WLMS NPs were acquired with a Bruker Minispec 60 (Karlsruhe, Germany) working at a Larmor frequency of 60 MHz (1.41 T) at 37 °C. The longitudinal relaxivity *r*_1_ and transverse relaxivity *r*_2_ values were calculated according to the general equation of relaxivity: *R* = *R*_0_ + *r* × [Fe in IO@SiO_2_] where *R* is the relaxation rate (1/*T*) in the presence of the core-shell IO@SiO_2_ nanoparticles, *R*_0_ the relaxation rate of the aqueous medium (in the absence of the NPs) and *r* the relaxivity value of the core-shell IO@SiO_2_.

#### 3.4.5. In Vitro Phantom Images

In vitro MRI-phantoms images were obtained on a clinical MRI (GE signa HD × t 3T, GE Healthcare, Milwaukee, WI, USA) running with a magnetic field of 3 T. T_1_ images were acquired with a spin echo sequence (TR = 400 ms, TE = 10 ms) and the T_2_ images were acquired with a fast spin echo sequence (TR = 2 s, TE = 52 ms).

#### 3.4.6. Magnetothermal Experiments and Specific Absorption Rate (SAR) Determination

The SAR measurements were obtained through a calorimetric method from a DM 100 instrument and DM applicator (Nanoscale Biomagnetics™, nB) under MaNIaC software (Nanoscale Biomagnetics™, nB, Zaragoza, Spain). Vials adapted for magnetothermal measurements and filled with 1.0 mL of the samples (IO@MS) at different iron concentrations were submitted to alternating magnetic fields (536.5 kHz; 300 Gauss). The increase of temperature was recorded for 10 min. A second-order polynomial function was used to fit the plot and to determine [dT/dt]_t = 0_ as described by Perigo et al. [43] to finally calculate the SAR value by using the following equation:

$$\mathrm{SAR}=m_{s}\times \frac{C_{s}}{m_{\mathrm{Fe}}}\times {\left[\frac{\mathrm{dT}}{\mathrm{dt}}\right]}_{t=0}$$

where m_s_ and C_s_ are respectively the mass (kg) and the heat capacity (J kg^−1^ K^−1^) of the sample, m_Fe_ (g) is the mass of iron element present in the sample and (dT/dt)*_t_*_= 0_ the derivative function of the temperature at t = 0 (K.s^-1^).

## 4. Conclusions

In this work, we aimed at exploring a new way to tune pore morphology and the resulting structural/textural features of MS NPs or MS shell around iron oxide magnetic cores. We showed here that the resulting pore morphology of MS NPs, synthesized with CTA^+^, Tos^−^ as pore structuring agent, AHMPD as a small organic amine basis acting as growth inhibitor and TEOS as sol gel silica source, can be tuned on a controlled manner simply by adjusting the sol gel reaction temperature T_sg_. Hence, we showed that below 75–80 °C, stellate MS NPs are formed as described in our previous works whereas above 80 °C, the morphology of the pore structure completely changes to a WL structure as observed by TEM and analyzed by nitrogen adsorption desorption isotherms. This process allowing to orient MS pore structure by simply adjusting the sol gel temperature is also successfully achieved around IO NPs yielding IO@STMS having a porous silica shell with stellate morphology when sol gel temperature is below 80 °C. IO@WLMS having a porous silica shell with WL morphology are obtained morphology with a temperature above 80 °C. The relaxometric and magnetothermal properties of the resulting core-shell magnetic silica IO@STMS and IO@WLMS displayed for both core shell NPs suitable features for T_2_-MRI and hyperthermia treatments. IO@STMS as compared to IO@WLMS, is shown to have higher transversal relaxivity r_2_ (respectively 307 vs. 156 mM^−1^·s^−1^) and slightly higher SAR measurements (ca. 500 W/g depending on the concentration), which can be explained by a better accessibility of water within the stellar pore structures. The large pore STMS shell around IO core ensure probably a better transversal relaxation of water and a slightly better heat dissipation to the solution. These results indicate that controlling the pore morphology, while controlling interaction with solvent, surface functionalities and other properties, are essential for the design of suitable nanomaterials envisioned for combined imaging and therapy applications.

## Figures and Tables

**Table 1:** Textural properties of the samples.

Sample	S_BET_, m^2^ g^−1^	V ^¤^_micro_, cm^3^ g^−1^	V ^§^_meso_, cm^3^ g^−1^	V_meso/_V_micro_	D_p-micro_, nm	D_p-meso_, nm
STMS50	713	0.08	0.53	6.6	≤0.5	10
WLMS80	951	0.11	0.97	8.8	≥0.8	4

^¤^ Integrated volume of adsorbed nitrogen from 0.5 to 0.8 nm from Horvarth-Kawazoe differential pore volume plot; ^§^ Integrated volume of adsorbed nitrogen from 2 to 15 nm from BJH desorption pore volume plot.

**Table 2:** Longitudinal and transversal relaxivities r_1_ and r_2_ for IO@WLMS and IO@STMS.

	r_1_ (mM^−1^·s^−1^)	r_2_ (mM^−1^·s^−1^)	r_2_/r_1_
IO@STMS	4.57	307	67
IO@WLMS	0.38	156	410

## Data Availability

The data presented in this study are available in this article.

## Supplementary Materials

The following are available online, Figure S1. Evolution of the MSN diameter with the sol-gel reaction temperature.

## Sample Availability

Not available.
