# Supplementary material for: Orienting the Pore Morphology of Core-Shell Magnetic Mesoporous Silica with the Sol-Gel Temperature. Influence on MRI and Magnetic Hyperthermia Properties
Source: Molecules. 2021 Feb 12;26(4):971. doi: 10.3390/molecules26040971 (PMC7917716; doi:10.3390/molecules26040971)
Supplement: Supplementary file 1 [file molecules-26-00971-s001.pdf]

Supporting Information

Orienting the pore morphology of magnetic porous silica with sol-gel temperature. Influence on MRI and magnetic hyperthermia properties.

Alexandre Adam[1], Ksenia Parkhomenko[2], Paula Duenas Ramirez[1], Clémence Nadal[1], Geoffrey Cotin[1], Paul Emmanuel Zorn[3,4], Philippe Choquet[3,4,5,6], Sylvie Bégin-Colin[1], Damien Mertz[1] *

[1]Institut de Physique et Chimie des Matériaux de Strasbourg (IPCMS), UMR-7504 CNRS-Université de Strasbourg, 23 rue du Lœss, BP 34 67034, Strasbourg Cedex 2, France, §

[2]Institut de Chimie et Procédés pour l'Energie, l'Environnement et la Santé (ICPEES), UMR-7515 CNRS-Université de Strasbourg, 25 rue Becquerel, 67087 Strasbourg, Cedex 2, France

[3] Imagerie Préclinique—UF6237, Pôle d'imagerie, Hôpitaux Universitaires de Strasbourg, France

[4]Service de Radiologie 2, Hautepierre, Pôle d'imagerie, Hôpitaux Universitaires de Strasbourg, France

[5]Icube, équipe MMB, CNRS, Université de Strasbourg, Strasbourg, France

[6]Fédération de Médecine Translationnelle de Strasbourg, Faculté de Médecine, Université de Strasbourg, Strasbourg, France

*E-mail : damien.mertz@ipcms.unistra.fr

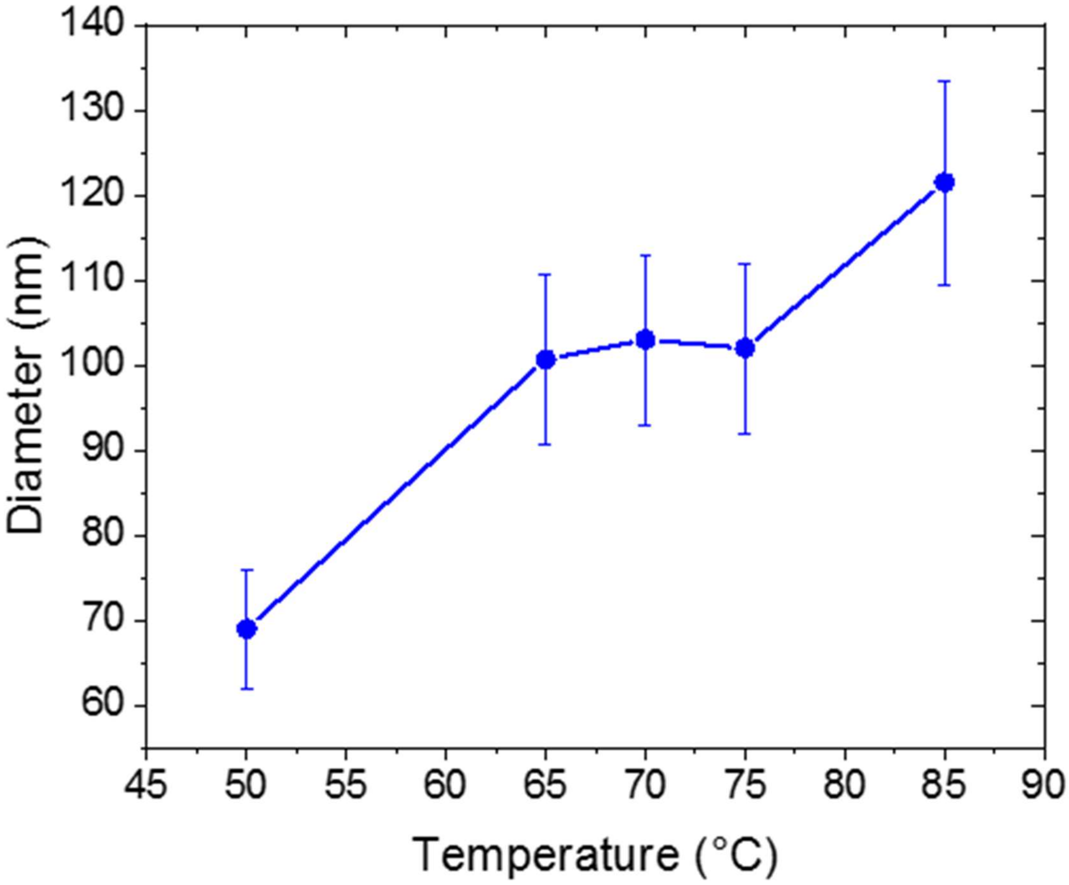

Figure S1. Evolution of the MSN diameter with the sol-gel reaction temperature.
